# Road Scene Simulation Based on Vehicle Sensors: An Intelligent Framework Using Random Walk Detection and Scene Stage Reconstruction

**DOI:** 10.3390/s18113782

**Published:** 2018-11-05

**Authors:** Yaochen Li, Zhichao Cui, Yuehu Liu, Jihua Zhu, Danchen Zhao, Jian Yuan

**Affiliations:** 1 School of Software Engineering, Xi’an Jiaotong University, No. 28 Xianning West Road, Xi’an 710049, Shaanxi, China; zhujh@mail.xjtu.edu.cn (J.Z.); yuanjian8868343@stu.xjtu.edu.cn (J.Y.); 2 Institute of Artificial Intelligence and Robotics, Xi’an Jiaotong University, No. 28 Xianning West Road, Xi’an 710049, Shaanxi, China; cui.zhichao@stu.xjtu.edu.cn (Z.C.); liuyh@mail.xjtu.edu.cn (Y.L.); danchenzhao@foxmail.com (D.Z.)

**Keywords:** road scene model, region detection, random walk, scene stage

## Abstract

Road scene model construction is an important aspect of intelligent transportation system research. This paper proposes an intelligent framework that can automatically construct road scene models from image sequences. The road and foreground regions are detected at superpixel level via a new kind of random walk algorithm. The seeds for different regions are initialized by trapezoids that are propagated from adjacent frames using optical flow information. The superpixel level region detection is implemented by the random walk algorithm, which is then refined by a fast two-cycle level set method. After this, scene stages can be specified according to a graph model of traffic elements. These then form the basis of 3D road scene models. Each technical component of the framework was evaluated and the results confirmed the effectiveness of the proposed approach.

## 1. Introduction

Road scene simulation and modeling based on vehicle sensors are currently an important research topic in the field of intelligent transportation systems [1,2]. The data acquired from vehicle-mounted sensors mainly include images, laser scans, and GPS. Once acquired, road scene modeling results can be applied to the simulation of photo-realistic traffic scenes.

A key use of such simulation is for the purposes of road safety, especially with regard to unmanned vehicles. Extensive tests and evaluations need to be performed to ensure the safety and robustness of unmanned vehicles. These mainly involve the field tests and offline tests. Field tests are the most traditional way to proceed. Micky Town at Michigan State and Google’s Castle Atwater in California have both been constructed to undertake unmanned vehicle evaluation. To be effective, field tests have to be implemented on different types of roads including urban roads, rural roads and highways. However, there are a number of drawbacks to using field tests, such as limited driving space, the time and effort involved in constructing them and their significant monetary cost. As a result, offline tests have become an increasingly important complement to field-based evaluation.

Early studies using offline testing were designed around computer graphics methods. Typical applications here included Prescan from TNO (Netherland) [3] and CarMaker from IPG (United States) [4]. Recently, road scene modeling based on road image sequences has become popular, for instance Google Street View [5] or Microsoft Street Slide [6]. These systems provide users with an immersive touring experience while browsing a map. The Waymo Team at Google (California, USA) have constructed a virtual scene platform from road image sequences that can be applied to the simulation of virtual roads for billions of miles.

In this paper, we propose a new framework for road scene simulation from road image sequences. This specific application we have in mind for this framework is the offline testing of unmanned vehicles. The framework consists of two successive steps: (1) the detection of road and foreground regions’ detection; and (2) the construction of scene models. For the first step, we have developed a region detection method based on a superpixel-level random walk (RW) algorithm, which is further refined by a fast two-cycle (FTC) level set approach. For the second step, foreground and background models are constructed using scene stages. “Floor-wall” structured traffic scenes are then established.

The main contributions of this work can be summarized as follows:

- The development of a novel region detection method using a superpixel RW algorithm. The superpixel features are computed using a combination of color, texture and location information. Region seeds for the RW algorithm are initialized through region-related trapezoids using an optical flow map. An FTC level set is then employed for region refinement.
- The development of a new framework for constructing 3D scene models based on scene stages. The scene stages are specified according to the detected road and foreground regions. 3D scene models are then constructed on the basis of graph models of the scene stages.
- The development of a new system to simulate traffic scenes. Two modes are designed for the simulation of traffic scenes: (1) an interactive touring mode, and (2) a bird’s-eye view mode. This system can form the basis of the offline testing of unmanned vehicles.

## 2. Related Works

The framework of this paper consists of several components, which mainly includes region detection and scene model construction. In this framework, the computation of the superpixel features is an important pre-processing step. A new RW algorithm is then proposed based on the superpixel features. The road scene models are further constructed based on the region detection results. The related works are summarized in the areas of region detection and scene model construction.

Superpixels are the mid-level processing units widely used in the computer vision community. Schick et al. [7] convert the pixel-based segmentation into a probabilistic superpixel representation. A Markov random field (MRF) is then applied to exploit the structural information and similarities to improve the segmentation. Lu et al. [8] specify the superpixel-level seeds using an unsupervised learning strategy. The appropriate superpixel neighbors are further localized by a GrowCut framework. However, the vanishing points in the images must be detected as a pre-processing step. Siogkas et al. [9] propose a RW algorithm integrating spatial and temporal information for road detection. Nevertheless, this pixel-level RW algorithm has the drawback of low efficiency and slow speed. Shen et al. [10] present an image segmentation approach using lazy random walk (LRW). The LRW algorithm with self-loop has the merits of segmenting the weak boundaries. However, this method cannot be applied to image sequences since the spatio-temporal superpixel features are not considered. The similar studies include the fixation point-based segmentation [11], etc. Recently, the region detection methods based on convolutional neural networks (CNN) become popular. Teichmann et al. [12] apply deep CNN to jointly reason about classification, detection and semantic segmentation based on the KITTI road dataset [13]. The effectiveness of residual networks (ResNet) [14] and VGG [15] are also evaluated. However, the training of the networks is computationally expensive.

The 3D scene models can be constructed based on the region detection results. Saxena et al. [16] propose a method to learn plane parameters using an MRF model. The location and orientation of each mesh facet can be judged to construct the 3D wireframe of the scene model. However, this method lacks a hypothesis about the scene layout, and the computation of the image features is time-consuming. Hoiem et al. [17] apply superpixel segmentation to the input images, and then utilize a support vector machine (SVM) for superpixel clustering. Delage et al. [18] apply a dynamic Bayesian network to automatically judge the edge pixels between the floor and wall regions for indoor images. The “floor-wall”-structured scene model is then constructed. Unfortunately, this scene model cannot be applied to the outdoor images. The Tour into the Picture (TIP) model of Horry et al. [19] partitions an input image into regions of “left wall”, “right wall”, “back wall”, “ceiling” and “floor”. The foreground objects are assumed to stand perpendicularly to the floor plane. The main drawback of the TIP model lies in the fact that it is applicable to the curved floor conditions. Nedovic et al. [20] introduce the typical 3D scene geometries called stages, each with a unique depth profile. The stage information serves as the first step to infer the global depth. Lou et al. [21] propose an approach that first predicts the global image structure, and then extract the pixel-level 3D layout. However, the prediction of the scene stage label is complex and computationally expensive.

The comparison of the previous studies is shown in Table 1, where region detection and scene construction are summarized, respectively.

The rest of the paper is organized as follows: The superpixel-level RW method and FTC refinement is presented in Section 3. In Section 4, the construction of road scene models is introduced. Experiments and comparisons are shown in Section 5. Finally, we close this paper with conclusion and future works.

## 3. Random Walk for Region Detection at a Superpixel Level

**Figure 1:**
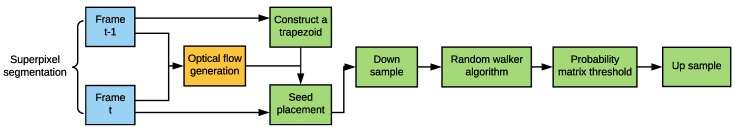
Flow diagram of the superpixel-level random walk algorithm at frame *t*.

In order to reconstruct road scene models, the road and foreground regions in each frame have to be detected first of all. To do this, we use a region detection method based on an RW algorithm. The road and the foreground regions of the first frame are specified by means of user annotation. For the rest of the frames, corresponding regions are detected based on the seeds propagated from the previous frames. The detection flow diagram for an example frame *t* is shown in Figure 1.

### 3.1. Superpixel Segmentation

For the region detection we use superpixel segmentation, specifically adopting the Ncut method [22], with superpixel features being extracted as the basis of the algorithm (see, Table 2). Color, texture and location descriptors are combined to denote the superpixel features. The color descriptor mainly used the HSV and CIElab color spaces. The texture descriptor is composed of a Gabor filter and a local binary pattern (LBP). The location descriptor consists of a convex hull, a clique number, etc. These descriptors are then concatenated into an overall vector.

### 3.2. Definition of Seed Trapezoid

**Figure 2:**
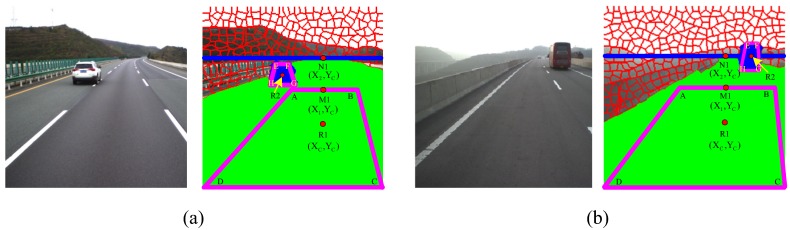
Definition of seed trapezoid for road and foreground regions. The green and blue colors denote the respective regions.

Seed trapezoids for the road and foreground regions are depicted in Figure 2. If R1 is taken to be the center of the detected road region of frame t−1, N1 can be denoted as the upper bound, with the same vertical coordinate as the centroid. The bottom line of the trapezoid (CD) is the same as frame t−1’s road base. The top of the trapezoid (AB) is equal to the perpendicular distance for R1N1. The seed trapezoid seed for the road region can then be specified. The trapezoid for the foreground region is defined in a similar way, as shown with regard to the placement in Figure 2.

### 3.3. Seed Trapezoid Initialization by Optical Flow

**Figure 3:**
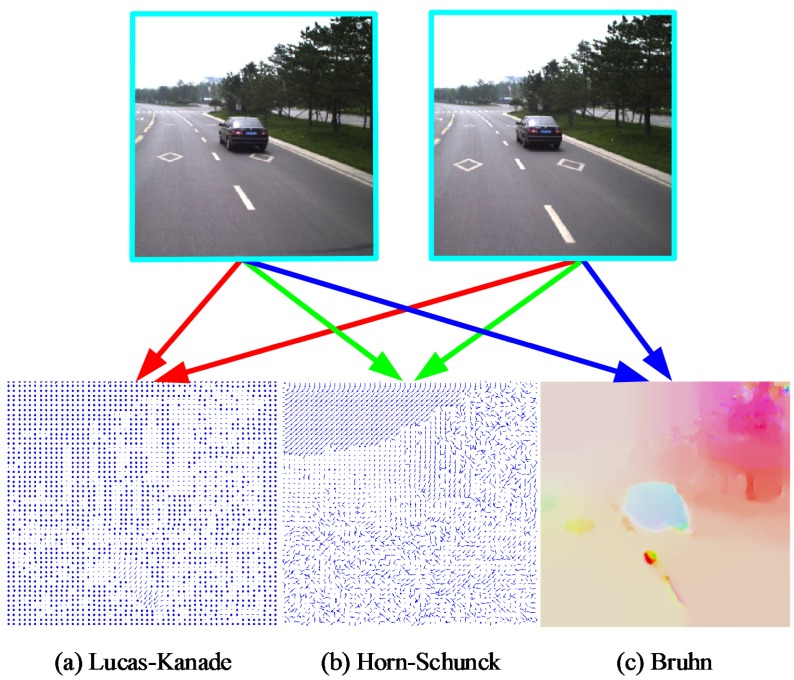
Optical flow map computed by different methods. (**a**) Lucas–Kanade; (**b**) Horn–Schunck; (**c**) Bruhn.

The trapezoid of a certain frame *t* is initialized in frame t−1 using an optical flow map. Optical flow map generation is typically classified into (1) sparse optical flow and (2) dense optical flow. Sparse optical flow is computed from the local feature points in images, e.g., by using the Lucas–Kanade algorithm [23]. Dense optical flow, however, attempts to compute the optical flow information for all of the pixels in an image, for instance, by using the Horn and Schunck [24] approach. The Bruhn method [25] brings together the advantages of both sparse and dense optical flows. A dense optical flow field is generated by using this method that is robust under conditions of environmental noise. A comparison of the results produced by the Lucas–Kanade, Horn–Schunck and Bruhn methods are shown in Figure 3.

### 3.4. Random Walk Detection at a Superpixel Level

The superpixel-level RW detection is shown in Algorithm 1. Its basic steps are as follows:

- The input image is transformed into the graph: G=(V,E), where the graph nodes denote the superpixels. The weight of the edges in the graph are defined as follows:
  (1) $$w_{ij}=\exp\left(-\beta {∥s_{i}-s_{j}∥}_{2}-{∥h_{i}-h_{j}∥}^{2}\right),$$
  where $s_{i}$ and $s_{j}$ are the features of superpixels *i* and *j*, while $h_{i}$ and $h_{j}$ denote the respective superpixel locations. The superpixel features are represented by a concatenation of the color and texture vectors:
  (2) $$s=[\underset{color}{\underset{︸}{R,\;G,\;B}},\;\underset{texture}{\underset{︸}{T_{1},\;T_{2},\;\ldots ,\;T_{N1}}}],$$
  where [R,G,B] denotes the color feature of the current superpixel. $\left[T_{1},T_{2},\ldots ,T_{N1}\right]$ denotes the texture feature, which is extracted by a Gabor filter. The image block centered at the current superpixel is used to compute the output vector of the Gabor filter. The superpixel features match those described in Section 3.1.
- A Laplacian matrix is now defined to denote the superpixel probability labels:
  (3) $$L_{ij}=\left\{\begin{array}{c} d_{i},\;\;\;\;\;\;\;\;\;\;\;\;\;\;\;\;if\;\;\;\;\;v_{i}=v_{j},\\ -w_{ij,}\;\;\;if\;\;\;\;\;\;v_{i}\;\;\;and\;\;\;v_{j}\;\;\;are\;\;\;adjacent\;\;\;nodes,\\ 0,\;\;\;\;\;\;\;\;\;\;\;\;\;\;\;\;otherwise, \end{array}\right.$$
  where $L_{ij}$ is the superpixel value indexed by $v_{i}$ and $v_{j}$, and $d_{i}=\sum w(e_{ij})$ is the degree for $v_{i}$ on all edges $e_{ij}$.
- Next, vertices of the graph *G* are partitioned into two groups: a seeded set $V_{M}$ and an unseeded set $V_{U}$. These two sets satisfy the following requirements: $V_{M}\cup V_{U}=V$ and $V_{M}\cap V_{U}=\emptyset$. The Laplacian matrix can now be redefined as:
  (4) $$L=\left[\begin{array}{cc} L_{M} & B_{d}\\ B_{d}^{T} & L_{U} \end{array}\right],$$
  where $L_{M}$ and $L_{U}$ are the seeded and unseeded pixels in *L* and $B_{d}$ is the decomposition result.
- The Dirichlet integral is:
  (5) $$D\left[x\right]=\frac{1}{2}x^{T}Lx=\frac{1}{2}\sum_{e_{ij}\in E}w_{ij}{\left(x_{i}-x_{j}\right)}^{2},$$
  where *x* is a function that minimizes Equation (5) and $e_{ij}$ is an edge of the graph connecting the vertices $v_{i}$ and $v_{j}$.
- The decomposition of Equation (5) can be defined as:
  (6) $$D\left[x_{U}\right]=\frac{1}{2}\left(x_{M}^{T}x_{U}^{T}\right)L\left[\begin{array}{c} x_{M}\\ x_{U} \end{array}\right],$$
  where $x_{M}$ and $x_{S}$ are the sets of regional probabilities corresponding to the unseeded and seeded superpixels. Differentiating $D[x_{U}]$ with respect to $x_{U}$ yields
  (7) $$L_{U}x_{U}=-B_{d}^{T}x_{M}.$$
  This equation can be solved using the method outlined in [9], with it being considered to be a two-class segmentation problem.

### 3.5. Region Refinement Using an FTC Level Set

The region detection algorithm described above is effective for most conditions. However, as the computing units of the algorithm are superpixels, there is a risk that, if the superpixel features are similar in the foreground and background regions, it may fail to get accurate results. To solve this problem, a fast two-cycle (FTC) level set method can be applied that is based on pixels for region refinement. The main data structure for this method is composed of three parts: an integer array ϕ as the level set kernel matrix, an integer array $F_{d}$ for the speed function and two linked lists of boundary pixels to denote the foreground contours $L_{in}$ and $L_{out}$. The foreground contours are initialized from the regions detected by the superpixel-level RW algorithm. In order to facilitate curve evolution of the FTC level set, the first cycle should be conducted to establish the data fidelity terms, then a second cycle should be conducted to establish the data smoothness terms. The pixels inside $L_{in}$ are called interior pixels, and the pixels outside $L_{out}$ are called exterior pixels. The kernel matrix can be defined as:

(8) $$\phi \left(y\right)=\left\{\begin{array}{c} 3\;\;\;\;\;\;\;\;\;\;\;\;\;\;\;\mathrm{if}\;\;\;y\;\;\;\mathrm{is}\;\;\;\mathrm{an}\;\;\;\mathrm{exterior}\;\;\;\mathrm{pixel},\\ 1\;\;\;\;\;\;\;\;\;\;\;\;\;\;\;\mathrm{if}\;\;\;y\;\in L_{out},\\ -1\;\;\;\;\;\;\;\;\mathrm{if}\;\;\;\;y\in L_{in}\;\;\;,\\ -3\;\;\;\;\;\;\;\;\mathrm{if}\;\;\;y\;\;\;\mathrm{is}\;\;\;\mathrm{an}\;\;\;\mathrm{interior}\;\;\;\mathrm{pixel}. \end{array}\right.$$

**Figure 4:**
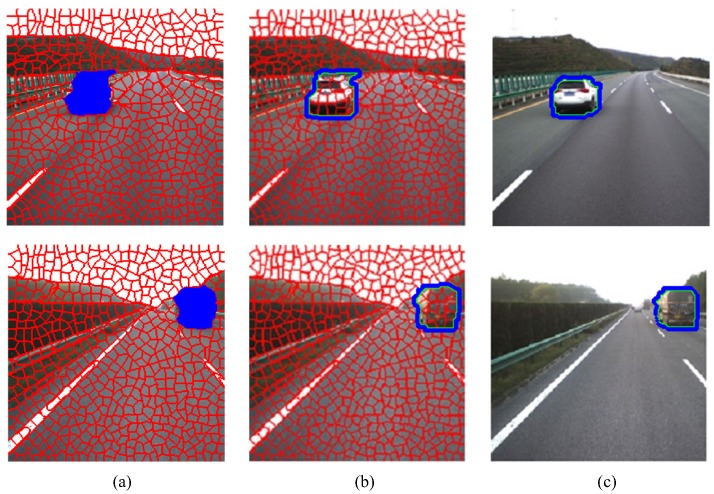
Region refinement based on the FTC level set. (**a**) the superpixel-level RW results; (**b**) the original region contours; (**c**) the refined region contours.

The speed function $F_{d}$ is defined according to the competition terms between the foreground and background regions [26]. The curve evolution can then be determined by using the signs of the speed function rather than by solving any partial differential equations. The speed function is as follows:

(9) $$F_{d}=\log\frac{p\left(f_{C}\left(y\right)|\Omega_{m}\right)p\left(f_{T}\left(y\right)|\Omega_{m}\right)}{p\left(f_{C}\left(y\right)|\Omega_{b}\right)p\left(f_{T}\left(y\right)|\Omega_{b}\right)},$$

where $\Omega_{m}$ and $\Omega_{b}$ are the feature pools for the foreground and the background, respectively. $f_{C}\left(y\right)$ and $f_{T}\left(y\right)$ are the color and texture feature vectors extracted at pixel *y*. After definition of the kernel matrix and speed function, the curve evolution can be implemented across two cycles. The first cycle is for the boundary pixel evolution. The second cyle is for the boundary smoothness. The functions switchIn() and switchOut() can be used for the curve evolution [27]. The region refinement examples based on the FTC level set are shown in Figure 4.

The region detection algorithm based on a superpixel-level RW with FTC refinement is summarized in Algorithm 1.

**Algorithm 1** Region detection based on a superpixel-level RW with FTC refinement
$\mathrm{Require}:\left\{\begin{array}{c} \text{Input image sequence}\;\left\{I_{1},\;\ldots ,\;I_{M}\right\};\\ \text{Superpixel set for each frame}\;\left\{S_{1},\;\ldots ,\;S_{M}\right\}; \end{array}\right.$
  1:Initialize the regions and trapezoids for the frame t=0;  2:(1) Region detection based on a RW at superpixel level:  3:**for** t = 1 to M **do**  4: Compute the optical flow map between frame *t* and t−1;   5: Initialize region seed of frame *t* using the trapezoid of frame t−1 on the basis of the optical flow map;  6: Down-sample frame *t* to a lower resolution;   7: Implement the RW algorithm for frame *t*;  8: Implement the threshold for the probability matrix to get the detection results;  9: Up-sample frame *t* to the original resolution; 10:
**end for**
 11:(2) Region refinement based on the FTC level set:  12:**for** t = 1 to M **do** 13: Initialize $L_{in}$ and $L_{out}$ based on the RW detection results;  14: Define the speed function $F_{d}$ using Eq. (9); 15: Undertake the Fast Two-Cycle curve evolution; 16: Get the refined regions according to the contours; 17:
**end for**

$\mathrm{Ensure}:\left\{\begin{array}{c} \text{The region detection results for the image frames}\;\left\{R_{1},\ldots R_{M}\right\};\\ \text{The seed trapezoid for each image frame}. \end{array}\right.$


## 4. Road Scene Construction and Simulation

After detection of the road and foreground regions, the corresponding static and dynamic traffic elements can be extracted. The scene models are then constructed based on the scene stages. These form the basis of the road scene simulation process.

### 4.1. Scene Model Construction

In order to construct the road scene models, a graph model can be used for each image to represent the connection between the foreground and background regions:

(10) G=(N,E),

where *N* and *E* represent the scene nodes and the corresponding relationships. For the *i*th image, $N_{i}$ and $E_{i}$ are defined by:

(11) $$\begin{array}{c} N_{i}=\left\{RP_{i},LW_{i},RW_{i},BW_{i},FG_{1},FG_{2},\ldots ,FG_{n}\right\},\\ E_{i}=\left\{LCPoints_{i},RCPoints_{i},PFG_{1},PFG_{2},\ldots ,PFG_{n}\right\}, \end{array}$$

where $RP_{i}$, $LW_{i}$, $RW_{i}$, and $BW_{i}$ denote “road plane”, “left wall”, “right wall” and “back wall”, respectively. $LCPoints_{i}$ and $RCPoints_{i}$ are the control points of road boundaries on each side [1]. $\left\{FG_{1},\ldots ,FG_{n}\right\}$ denotes the *n* foreground objects, while $\left\{PFG_{1},\ldots ,PFG_{n}\right\}$ are the positions of rht foreground polygons standing on the road plane.

**Figure 5:**
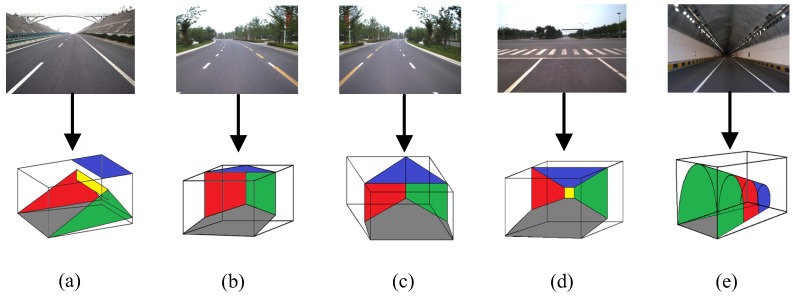
Scene stages of traffic scenes. (**a**) through lane; (**b**) left-turn lane; (**c**) right-turn lane; (**d**) intersection; (**e**) tunnel.

A set of scene stages STG is defined in advance, which can represent the basic structures of typical road scenes. A related scene stage can be specified for each graph model to denote the scene’s geometric structure. The scene stage is a traffic scene wireframe model that represents the scene layout for 3D traffic scene modeling. Scene stages can be classified into simple and complex types, as shown in Figure 5. A simple scene stage consists of just the background elements of a traffic scenes. A complex scene stage consists of both the background and foreground elements.

For the simple scene stages, the corresponding background models have a “floor-wall” structure, with the road region being located on the horizon plane. The rest of the background regions are assumed to be perpendicular to the road plane. For the complex scene stages, the foreground models are constructed from rectangular polygons, which are set vertically to the road plane. The foreground polygons have RGBA data structure, with *A* denoting the transparency ratio. The polygons in the region outside of the foreground are set to be transparent.

**Figure 6:**
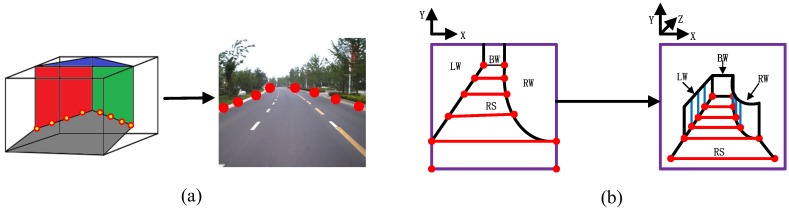
Generation of control points. **(a)** labeling of control points; (**b**) scene modeling results.

We determine the control points for the road boundaries according to the traffic scene wireframe model. The control points are distributed on both sides of the road. In each scene stage, the two farthest control points are taken from the far end of the road surface, while the two closest control points are specified by the near end of the road surface. The remaining control points are distributed between the farthest and the nearest control points, as shown in Figure 6a. A 3D corridor road scene model can then be constructed according to the control points (this principle is shown in Figure 6b). In addtion to traditional scene models, cartoon scene models can be constructed by applying non-photorealistic rendering to the input images [28]. The road scene model construction process is summarized in Algorithm 2.

**Algorithm 2** Road scene model construction.**Require:** The region detection results for the image frames $\left\{R_{1},\ldots R_{M}\right\}$.
1:**for**t=1 to M **do**2: Specify the foreground regions $\left\{F_{1}^{t},F_{2}^{t},\ldots ,F_{n}^{t}\right\}$ and road region $RD^{t}$ from $R_{t}$;3: Construct the scene graph model G=(N,E);4: Specify the scene stage $STG^{t}$ according to the scene graph model *G*;5: Perform the scene construction based on the geometric structure of $STG^{t}$.6:**end for**
**Ensure:** Road scene models with the “floor-wall” structure.

### 4.2. Road Scene Simulation

**Figure 7:**
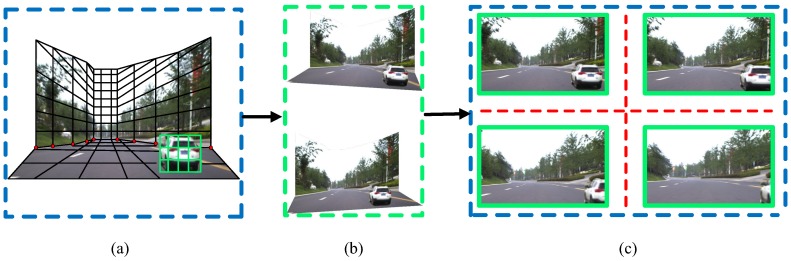
New viewpoint images rendering. (**a**) the 3D traffic scene model. The black mesh denotes the background scene, while the green mesh denotes the foreground vehicle; (**b**) the control of view angles; (**c**) the new viewpoint images.

The 3D traffic scene simulation can be performed by assembling the “floor-wall”-structured scene model. The viewpoint can be freely changed during the simulation process by using the commands forward, back, up and down. With the assistance of GPS data, the trajectory of the viewpoint can be displayed on the map. Depending on how the viewpoint moves, the traffic scene simulation can be classified into two modes: (1) Roaming mode, where new viewpoint images are generated according to the movement of the viewpoint; (2) Bird’s-eye view mode, where the viewpoint is static and virtual vehicles and obstacles can be added into the scene. The generation of new viewpoint images is shown in Figure 7a. The black grid represents the basic scene model. The green grid denotes the foreground vehicle model. Users can change the viewing angle through adjustments of the road scene model. The new viewpoint images are then generated, as shown in Figure 7b.

We have designed five metrics for the offline testing of unmanned vehicles, namely: pedestrian recognition; collision avoidance; traffic signal recognition; pavement identification; and fog recognition. Four levels of performance for each metric were also defined to evaluate vehicle behavior, (L0, L1, L2, L3), as shown in Table 3. The complexity of the road scene can generally be divided into three categories, (R1, R2, R3), according to: road conditions; special areas; and special kinds of weather. On the basis of these categories, various scenes can be constructed easily for the offline testing of unmanned vehicles. The three categories of road complexity are as follows:

- R1: Different road conditions including rural roads, urban roads, highways and tunnels. The road parameters relate to the specific road width, the number of obstacles, traffic signs and traffic lights.
- R2: Special areas including campuses, hospitals, crowded streets, etc. Vehicles in special areas need to react quickly and perform different operations.
- R3: Special kinds of weather including rain, snow, fog, etc. The special kinds of weather may also be of varying degree. This needs to be accurately identified for the evaluations.

Finally, the performance of unmanned vehicles may be evaluated across a combination of different degrees of scene complexity and different evaluation metrics.

## 5. Experiments and Comparisons

Our experiments were undertaken on a computer with an Intel i5 processor @3.33 GHZ and with 16 GB Memory). The experimental data was mostly taken from the TSD-max dataset [29], which was constructed by the Institute of Artificial Intelligence and Robotics at Xi’an Jiaotong University in China. The dataset is composed of road images captured from urban roads, rural roads, highways, etc. Specifically, the experiment of road region detection is also based on the KITTI dataset [12].

### 5.1. Evaluation of the Region Detection

**Figure 8:**
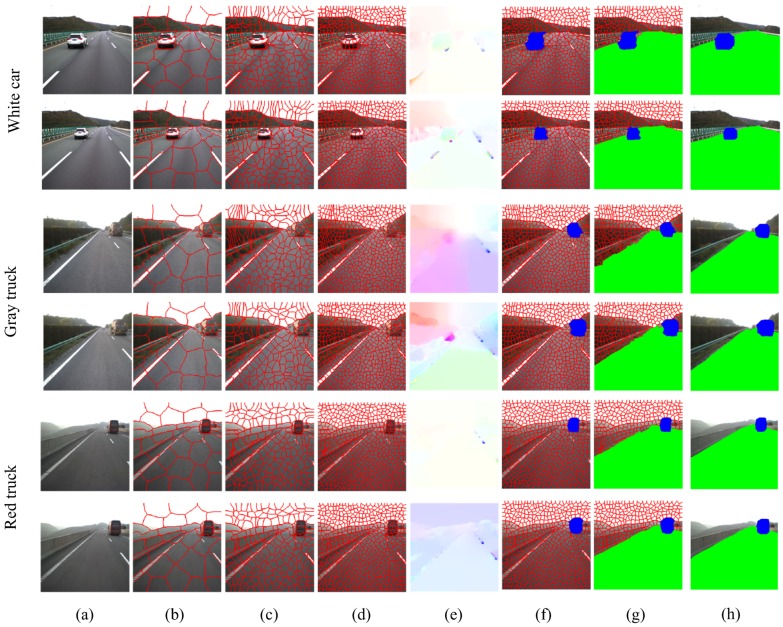
Region detection results based on the RW method (TSD-max dataset). (**a**) input image sequence; (**b**–**d**): superpixel segmentation with superpixel numbers of *N* = 40, *N* = 200, and *N* = 500, respectively. (**e**) optical flow map between adjacent frames; (**f**) detection of foreground regions; (**g**) detection of both foreground and road regions; (**h**) region refinement by FTC level set.

**Figure 9:**
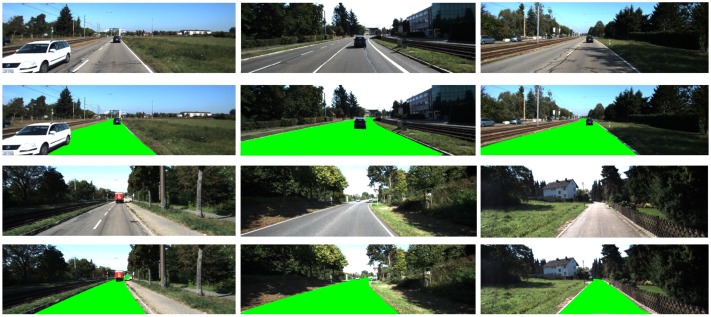
Road region detection results based on an RW method with refinement (KITTI dataset).

First of all, we perform the region detection experiments. Three image sequences are selected for the region detection experiments: White Car (512 × 512 pixel size, 200 frames), Gray Truck (512 × 512 pixel size, 300 frames), and Red Truck (512 × 512 pixel size, 250 frames). The KITTI dataset is utilized for the task of road region detection (1242 × 375 pixel size, 200 frames). Figure 8 shows the superpixel segmentation at different scales, the optical flow map between adjacent frames, and the region detection results before and after refinement. The road detection results based on the superpixel-level RW method with refinement for the KITTI dataset is shown in Figure 9. The algorithms mainly work on the basis of a superpixel segmentation size of N=500. For quantitative evaluation, we use three metrics: precision; recall; and F-measure to compare with the ground truth road and foreground regions. Precision denotes the ratio of correct pixels within the detected road region. Recall denotes the ratio of correct pixels in relation to the benchmark road region. In our experiments, precision and recall are defined as follows:

(12) $$\begin{array}{c} Pre=\frac{\left|R\cap R_{G}\right|}{R},\;\;\;\;\;\;Rec=\frac{\left|R\cap R_{G}\right|}{R_{G}}, \end{array}$$

where *R* and $R_{G}$ denote the detected region and ground truth, respectively.

The *F-Measure* can be computed by combining *Pre* and *Rec*:

(13) $$\begin{array}{c} F-Measure=\frac{(1+\alpha )\cdot Pre\cdot Rec}{\alpha \cdot Pre+Rec}. \end{array}$$

We set α=0.5 to give precision and recall equal weight.

We employ the superpixel SVM [17], Markov random field (MRF) [7], GrowCut [8] and Siogkas’s RW [9] as baseline methods for road region detection. Besides these methods, VGG16 [15] and ResNet101 [14] are implemented for the comparison of road detection results. The average values for all of the image sequences are shown in Table 4 and Table 5. With regard to the methods presented in this paper, the superpixel-level random walk (SRW) and the superpixel-level RW with level set refinement (SRW+Refine) are implemented. As the results of the comparisons show, the accuracy our proposed method is approximately similar to ResNet101, which is superior to the other baseline methods.

### 5.2. Evaluation of the Scene Construction

**Figure 10:**
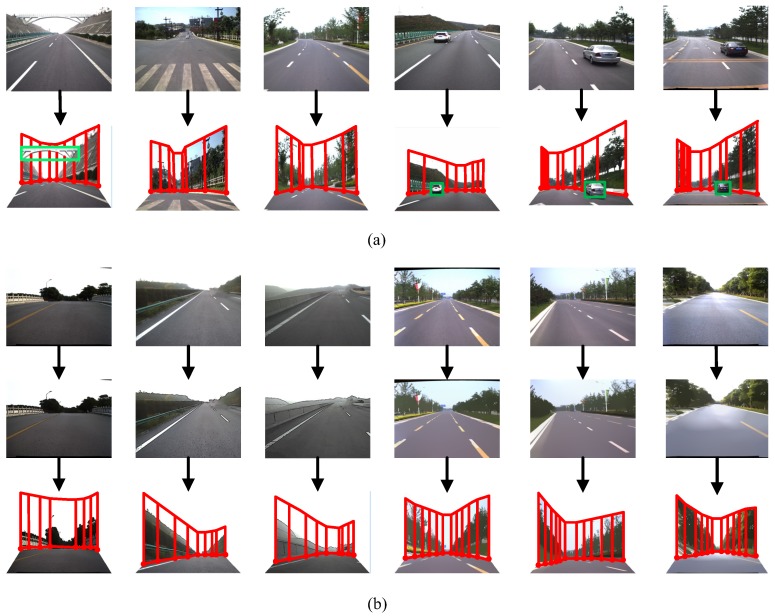
Construction of scene models. (**a**) traditional scene models; (**b**) cartoon scene models.

Next, we performed experiments relating to the scene construction and simulation. Input image sequences with a resolution of 1024 × 1024 pixels were once again selected from the TSD-max dataset, as shown in Figure 10. There were three main types of scene models: (1) simple scene models based on pure background images; (2) complex scene models based on both foreground and background images; and (3) cartoon scene models based on non-photorealistic images [28].

**Figure 11:**
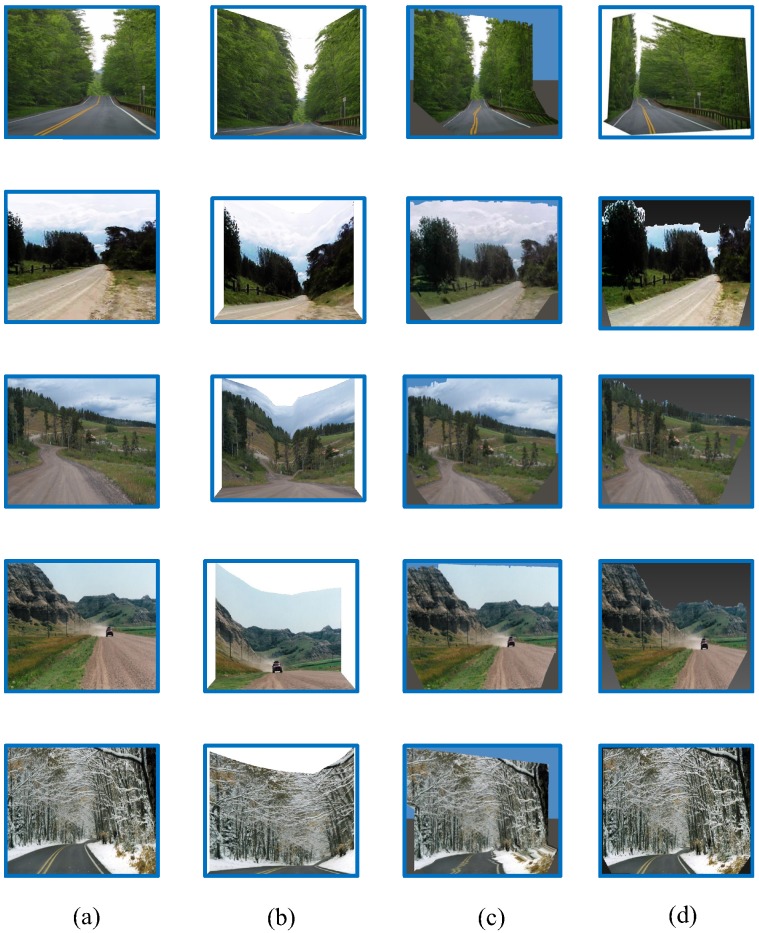
Comparison of 3D traffic scene construction methods. (**a**) input images; (**b**) proposed method; (**c**) Make3D; (**d**) photo pop-up.

The scene models generated by our own approach were compared to those generated by Make3D [16] and Photo Pop-up [17], as shown in Figure 11. The results demonstrate that distortions can occur in the Make3D and Photo Pop-up models. When compared to these baseline methods, our own approach produce the most realistic results. We also find that our proposed scene structure is more suitable for curved road conditions.

To qualitatively evaluate the scene models, we apply two metrics [16]: (1) a plane correctness ratio, with a plane being defined as correct if more than 75% of the plane patches are correctly detected as semantic wall and road regions; (2) a model correctness ratio, with a model being defined as correct if more than 75% of the patches in the wall and the road planes had the correct relationship to their neighbors. The evaluation is performed by someone who is not associated with the project. One-thousand road images with a resolution of 1024 × 1024 are chosen overall. The comparative results according to these metrics are shown in Table 6. As can be seen, the proposed scene models outperform the baseline methods in terms of both plane and model correctness.

## 6. Conclusions and Future Works

In this paper, we have proposed a framework for road scene model construction via superpixel-level RW region detection. The RW detection is able to locate road and foreground regions simultaneously. After segmentation of the superpixels, a region seed for the first frame is specified through user annotation. For each subsequent frame, the region seeds are located using trapezoids propagated from the previous frame. The RW algorithm is implemented through initialization of the region seeds, with the superpixels being utilized as graph nodes. In order to achieve more accurate region detection results, an FTC level set algorithm was also implemented for region refinement. After detection of the road and foreground regions, 3D corridor-style scene models can be constructed, depending on the type of scene stage. 3D traffic scene simulations can then be developed that are based on the scene models.

In the future research, we intend to use depth maps to construct more detailed scene models. A multi-view registration of point clouds can then be implemented to reconstruct 3D foreground structures. Cuboid models of the traffic elements can also be constructed on the basis of this for an even more sophisticated simulation of traffic scenes.

## Figures and Tables

**Table 1:** Overview of the previous studies.

**Region Detection**		Lu’s [8]	Siogkas’ [9]	Teichmann’s [12]	ResNet101 [14]	Ours
	Superpixel pre-processing	Yes	Yes	No	No	Yes
	Spatio-temporal features	No	Yes	No	No	Yes
	For image Sequence	Yes	No	Yes	Yes	Yes
**Scene Construction**		Horry’s [19]	Saxena’s [16]	Hoiem’s [17]	Lou’s [21]	Ours
	“floor-wall” geometry	Yes	Yes	Yes	No	Yes
	Scene stage	No	No	No	Yes	Yes
	For image sequence	No	No	No	No	Yes

**Table 2:** Feature descriptors of superpixels (superpixel cliques).

Feature Descriptors	Feature Numbers
**Color**	**9**
C1 RGB color	3
C2 HSV color	3
C3 CIELAB	3
**Texture**	**62**
T1 Gabor filters: 4 scales, 6 orientations	48
T2 Local binary pattern: 3 × 3 template	9
T3 Edge histogram descriptors	5
**Locations and Shapes**	**6**
L1 Location: Normalized *x* and *y* coordinates	2
L2 Shapes: Superpixel number in the clique	1
L3 Shapes: Edge number within convex hull	1
L4 Shapes: Ratio of the pixels to the convex hull	1
L5 Shapes: Whether the clique is continuous	1

**Table 3:** Evaluation metrics of unmanned vehicles.

	L0	L1	L2	L3
Pedestrian detection	Partially detect	Mostly detect	Fully detect	Perfectly detect
collision avoidance	Fully collide	Always collide	Partially collide	Perfectly avoid
Traffic signal recognition	Partially identify	Mostly identify	Fully identify	Perfectly identify
Pavement identification	Not at all	Misidentify	Recognize	Perfectly recognize
Rain and fog identification	Not at all	Misidentify	Recognize	Perfectly recognize

**Table 4:** Evaluation of road detection results.

Datasets	Methods	Precision	Recall	F-Score
White Car	SVM [17]	0.8650	0.8720	0.8673
	MRF [7]	0.9040	0.9150	0.9076
	GrowCut [8]	0.9280	0.9320	0.9232
	Siogkas [9]	0.9350	0.9410	0.9370
	VGG16 [15]	0.9425	0.9500	0.9450
	ResNet101 [14]	0.9480	**0.9560**	0.9507
	SRW	0.9400	0.9420	0.9407
	SRW+Refine	**0.9520**	0.9550	**0.9530**
Gray Truck	SVM [17]	0.8550	0.8680	0.8593
	MRF [7]	0.9180	0.9100	0.9153
	GrowCut [8]	0.9220	0.9280	0.9240
	Siogkas [9]	0.9300	0.9330	0.9310
	VGG16 [15]	0.9355	0.9500	0.9403
	ResNet101 [14]	**0.9480**	0.9400	**0.9453**
	SRW	0.9350	0.9400	0.9367
	SRW+Refine	0.9460	**0.9420**	0.9447
Red Truck	SVM [17]	0.8700	0.8750	0.8717
	MRF [7]	0.9120	0.9050	0.9097
	GrowCut [8]	0.9300	0.9360	0.9320
	Siogkas [9]	0.9380	0.9420	0.9393
	VGG16 [15]	0.9290	0.9350	0.9310
	ResNet101 [14]	**0.9450**	0.9420	0.9440
	SRW	0.9350	0.9400	0.9367
	SRW+Refine	0.9440	**0.9480**	**0.9453**
KITTI	SVM [17]	0.7900	0.7520	0.7769
	MRF [7]	0.8250	0.8330	0.8276
	GrowCut [8]	0.8580	0.8930	0.8694
	Siogkas [9]	0.8895	0.9050	0.8946
	VGG16 [15]	0.9110	0.9050	0.9090
	ResNet101 [14]	0.9350	0.9225	0.9308
	SRW	0.9220	0.9155	0.9198
	SRW+Refine	**0.9360**	**0.9350**	**0.9357**

**Table 5:** Evaluation of foreground detection results.

Datasets	Methods	Precision	Recall	F-Score
White Car	SVM [17]	0.825	0.865	0.8379
	MRF [7]	0.890	0.902	0.8940
	GrowCut [8]	0.9125	0.9290	0.9179
	Siogkas [9]	0.9315	0.9350	0.9327
	SRW	0.9400	0.9280	0.9360
	SRW+Refine	**0.9480**	**0.9575**	**0.9511**
Gray Truck	SVM [17]	0.8570	0.8785	0.8640
	MRF [7]	0.8580	0.8690	0.8616
	GrowCut [8]	0.9155	0.9225	0.9178
	Siogkas [9]	0.9280	0.9310	0.9290
	SRW	0.9305	0.9320	0.9310
	SRW+Refine	**0.9385**	**0.9490**	**0.9420**
Red Truck	SVM [17]	0.8660	0.8725	0.8682
	MRF [7]	0.8610	0.8650	0.8623
	GrowCut [8]	0.9235	0.9320	0.9263
	Siogkas [9]	0.9345	0.9380	0.9357
	SRW	0.9390	0.9410	0.9397
	SRW+Refine	**0.9455**	**0.9520**	**0.9477**

**Table 6:** Comparisons of scene models.

	Make3D [16]	Photo Pop-Up [17]	Proposed
Correct Planes	87%	78%	**94%**
Correct Models	90%	92%	**96%**
